# A Method on Dynamic Path Planning for Robotic Manipulator Autonomous Obstacle Avoidance Based on an Improved RRT Algorithm

**DOI:** 10.3390/s18020571

**Published:** 2018-02-13

**Authors:** Kun Wei, Bingyin Ren

**Affiliations:** School of Mechatronics Engineering, Harbin Institute of Technology, Harbin 150001, China; weikun@hit.edu.cn

**Keywords:** RRT algorithm, robotic manipulator, dynamic unstructured environment, autonomous obstacle avoidance, dynamic path planning

## Abstract

In a future intelligent factory, a robotic manipulator must work efficiently and safely in a Human–Robot collaborative and dynamic unstructured environment. Autonomous path planning is the most important issue which must be resolved first in the process of improving robotic manipulator intelligence. Among the path-planning methods, the Rapidly Exploring Random Tree (RRT) algorithm based on random sampling has been widely applied in dynamic path planning for a high-dimensional robotic manipulator, especially in a complex environment because of its probability completeness, perfect expansion, and fast exploring speed over other planning methods. However, the existing RRT algorithm has a limitation in path planning for a robotic manipulator in a dynamic unstructured environment. Therefore, an autonomous obstacle avoidance dynamic path-planning method for a robotic manipulator based on an improved RRT algorithm, called Smoothly RRT (S-RRT), is proposed. This method that targets a directional node extends and can increase the sampling speed and efficiency of RRT dramatically. A path optimization strategy based on the maximum curvature constraint is presented to generate a smooth and curved continuous executable path for a robotic manipulator. Finally, the correctness, effectiveness, and practicability of the proposed method are demonstrated and validated via a MATLAB static simulation and a Robot Operating System (ROS) dynamic simulation environment as well as a real autonomous obstacle avoidance experiment in a dynamic unstructured environment for a robotic manipulator. The proposed method not only provides great practical engineering significance for a robotic manipulator’s obstacle avoidance in an intelligent factory, but also theoretical reference value for other type of robots’ path planning.

## 1. Introduction

In a future intelligent factory where the environment will be dynamic and unstructured, a robotic manipulator will work with humans efficiently and safely to complete a great variety of complex jobs and tasks collaboratively [1,2,3,4]. A collaborative manipulator firstly can perceive static obstacles and avoid obstacles autonomously. Research on autonomous obstacle avoidance for a robotic manipulator in a static environment has been given much more attention [5,6]. However, above all, a robotic manipulator must also avoid dynamic obstacles autonomously at the same time, such as a sudden entry from a human, which requires the manipulator to accomplish dynamic path planning [7].

Path planning is defined as that a non-collision continuous path for a robotic manipulator can be found from an initial pose to a target pose in a configuration space in which the manipulator’s constraints must be satisfied [8]. Among the path-planning methods, the Rapidly Exploring Random Tree (RRT) algorithm based on random sampling has been widely applied in dynamic path planning for a high-dimensional robotic manipulator in a complex environment. RRT has probability completeness, perfect expansion, and a fast exploring speed, so there is no need to map the obstacle from the task space to the configuration space. However, the existing RRT algorithm cannot well-resolve a path planning issue when the robotic manipulator is faced with a dynamic unstructured environment in which lots of obstacles are distributed randomly and non-uniformly. For instance, the slow node extension speed of the RRT algorithm reduces the rate of convergence, which fails to meet the real-time requirement of dynamic path planning for a robotic manipulator. Furthermore, the obstacle constraint makes the path generated by the RRT algorithm’s random sampling contain many unnecessary breakpoints, resulting in unsmooth and discontinuous curvature paths. Consequently, the motion tracked by the manipulator is often unstable.

For the above drawbacks and deficiencies of the traditional RRT, a set of improvements have been proposed in related literature by many researchers in recent years. For exploring the speed problem, the Bi-RRT algorithm was proposed by Kuffner and LaValle [9] in which two trees were grown up from the initial state and target state, respectively, thus improving the algorithm’s exploring and convergence speed. Then, RRT-connect was proposed to improve node extension efficiency [10]. A target directional exploring algorithm for a seven-degree of freedom (DOF) redundant manipulator was presented to accelerate path planning in the literature [11], whose shortcoming was that the exploring space must be changed sometimes in its practical application. A joint configuration space for a series manipulator was constructed via the traversing method and the RRT algorithm was utilized to explore the path without collision [12], whose drawback lay in the simplicity of the model of the manipulator and the obstacle according to their geometry features. A dynamic path-planning method based on Probabilistic Roadmap (PRM) and RRT was presented when given a specific end effector task for a mobile manipulator, and was validated via a simulation to avoid static and dynamic obstacles efficiently, but was limited to simple cuboid and cylinder obstacles [13].

For the unsmooth path problem as a result of RRT randomness, Kuwatat [14] proposed a Dubins path consisting of the line and the arc, but the path curvature was not continuous. Fraichard and Scheuer [15] utilized a clothoid curve to smooth the path; however, it could not be obtained accurately in real time because of its non-closed form solution. Lau et al. [16] used a quintic Bezier curve, but path curvature continuity was not taken into consideration. Elbanhawi et al. [17] proposed a cubic *B* spline algorithm, which met the non-holonomic constraint requirement for a mobile robot with continuous curvature.

Therefore, in this paper, a dynamic path-planning method for robotic manipulator autonomous obstacle avoidance based on an improved RRT algorithm, called Smoothly RRT (S-RRT), is proposed to overcome the deficiencies reviewed above. A human-like entity model is used as the static obstacle while a real human arm is used as the dynamic obstacle. The contour information of the obstacle is obtained by a Kinect RGB-D sensor and point cloud post-processing. The target directional node extension method is firstly introduced, which can increase the RRT algorithm’s sampling speed dramatically. Then, a path optimization strategy based on the maximum curvature constraint is presented to generate a smooth and curved continuous executable path for a robotic manipulator. Finally, the correctness, effectiveness, and practicability of the proposed method are demonstrated via a MATLAB static simulation and an ROS (Robot Operating System) dynamic simulation as well as a real autonomous obstacle avoidance experiment in a dynamic unstructured environment for a robotic manipulator.

## 2. Improved RRT Algorithm

### 2.1. Traditional RRT Algorithm

Path exploring in high-dimension space is much more complex than that in low-dimension space. The basic idea of the RRT algorithm is to explore in the form of the tree from the initial part of the manipulator and to sample randomly in feasible space so as to extend the branches and leaves until the exploring tree covers the target region.

$T_{k}$, $q_{init}$, $q_{goal}$, and $q_{rand}$ represent the exploring random tree containing k nodes, the initial state, the target state, and choosing the state point randomly in the configuration space, respectively. The points are generated and the branches of RRT exploring trees are extended until the target *position* can be found. The pseudo codes of the RRT algorithm are the following:

Build_RRT(*q_init_*)

1. T.init(*q_init_*);
2. for *k* = 1 to *k* do
3. *q_rand_* ← Random_State()
4. Extend(*T*, *q_rand_*);
5. Return *T*

Traverse the random tree $T_{k}$ and find $T_{k}$, where means the nearest to leaf node $q_{near}$. $dist(q_{near},q_{rand})$ represents the scale function between two nodes in the configuration space, which denotes the distance between two nodes.

If $dist(q_{near},q_{rand})<L$, it will mean that the exploring tree has already extended the target region, else $dist(q_{near},q_{rand})\geq L$; and $q_{new}$ will be found on the connecting line between $q_{new}$ and $q_{rand}$ with $dist(q_{near},q_{rand})=\varepsilon$, where ε denotes exploring step length. If $q_{new}$ does not exceed the joint limit and has no collision with an obstacle, the exploring tree will increase by a new node, else it will regenerate randomly a new node $q_{rand}$. Repeat the above process until the target region is reached. The pseudo codes of the branch extension of an RRT exploring tree are the following:

Extend(*T*, *q*)

1. *q_near_*←Nearest_Neighbor(*q*, *T*);
2. If New_State(*q*, *q_near_*, *q_new_*, *u_new_*) then
3. T.add_vertex(*q_new_*);
4. T.add_edge(*q_near,_ q_new,_ u_new_*);
5. if *q_new_* *= q* then
6. Return Reached;
7. else
8. Return Advanced;
9. Return Trapped;

### 2.2. Node Extension

Node extension is the key step in RRT path exploring. The traditional algorithm adopts the pure random sampling method and extends toward the outside. The heuristic exploring method proposed in recent years has provided new ideas for path exploring. Target direction is a very important idea for RRT planning node extension. Extension toward the target position directly accelerates the RRT exploring process. However, in order to keep the randomness, it is necessary for some nodes to extend randomly to maintain a balance between random extension and target direction. The pseudo codes of the sampling process are the following:

Sample(*T*)

1. *p*←Random(0, 1.0)
2. if *p* < *P_goal_;*
3. Return goal;
4. else
5. Return RandomNode();

The target directional probabilistic threshold value $P_{goal}$ determines whether the RRT grows up toward the target point or randomly, which helps to find a feasible growing direction without exploring aimlessly. The objective of growing randomly is to keep the completeness of the RRT algorithm. To enhance target direction, the first target and then the randomness sampling method are adopted. The pseudo codes of the improved RRT node extension are the following:

Improve_Extend(*T*, *q*)

1. result←Extend(*T*, goal)
2. if Trapped = result
3. *q_rand_*←Random_Node()
4. while Trapped = Extend(*T*, goal)
5. Random_Extend(*T*, *q_goal_*);
6. else
7. Improve_Extend(*T*)

When the target directional extension encounters collision, the pseudo codes of the random extension strategy are the following:

Random_Extend(*T*, *q*)

1. *p*←Random(0, 1.0)
2. if *p* < *P_best_*
3. Extend(*T*, *q_nearest_*, *q*);
4. else
5. Extend(*T*, *q*);

The target direction of the improved extension method is more definite. The whole process will recurve until reaching the target region if no collision happens. If the target directional extension encounters collision, the random extension method will be adopted to escape from the collision region.

The random extension strategy includes a random extension of the whole tree and the point neighbor nearest to the target. The parameter $P_{best}$ is utilized to adjust their ratio. The random extension method is used until a feasible solution is found. $P_{best}$ in this paper is chosen to be 0.6.

### 2.3. Collision Inspection

Collision inspection is a very crucial part of the RRT algorithm, which is the main criteria to determine whether a sampling point can be feasible and is considerably time-consuming as well. The open source collision inspection library called the Flexible Collision Library [18] (FCL) provides the computation for object collision inspection and approaching distance. FCL can inspect the collision or distance of a traditional triangular surface and basic body shapes, such as a sphere, a cube, and a cylinder. Furthermore, FCL is also able to inspect the collision between the point clouds. Additionally, FCL has an advantage with high collision inspection efficiency and little time, which is very suitable for an ROS. Therefore, FCL was called to complete the collision inspection with the RRT algorithm in this paper. The reasonableness of the sampling points should be checked during the sampling process of RRT, including whether the joint angle is beyond the limitation and the manipulator encounters collision. It is simple to check the joint angle. The FCL library is applied to inspect the collision for the manipulator. The pseudo codes of collision inspection are the following:

Collision_Detection(*x*)

1. Forward_Kinematics(*x*);
2. for *k* = 1 to 6 do
3. *C_k_*←FCL_Cylinder_Create(*x*, *k*)
4. if FCL_Cylinder_Collision(*C_k_*)
5. Return Trapped;
6. Return Advanced;

The position and orientation of each link frame are computed via the forward kinematics of the manipulator to obtain the center position and axis orientation of six cylinders. The FCL library is used to check the collision between cylinders and surrounding obstacles one-by-one. The above process of collision inspection will be carried out again and repeatedly if a collision exists.

### 2.4. Trajectory Optimization

The path planned by the RRT algorithm is always not optimal as a result of its strong randomness. The generated paths are unsmooth with a discontinuous curvature that includes many unnecessary break points. Furthermore, the obstacle constraint contributes to the generation of break points, especially in a dynamic unstructured environment, which leads to instability in the manipulator’s ability to track the path and even results in destruction. Therefore, it is necessary to optimize the trajectory aimed at solving the path-planning problem for the manipulator in a complex environment. Smooth paths with a continuous curvature are generated in combination with the improved RRT algorithm. The pseudo codes of the trajectory optimization method are the following:

Trajectory Optimize(*T*)

1. *Q*←Pruning(*T*)
2. *Q*←Inser_MidNode(*Q*)
3. *S*←Cubic_Bspline(*Q*)
4. Return *S*

Function Pruning(*T*)

5. *T*←obtained from S-RRT
6. Var *Q*_1_, *Q*_2_: path
7. *Q*_1_ (*q*_0_, *q*_1_, *q*_2_, ⋯, *q*_n_) = Path(*T*)
8. *q_temp_*←*q*_0_; *Q*_2_.Add_Node(*q*_0_)
9. while *q_temp_*! = *q_n_* do
10. for each node *qi* ∈ *Q*_1_
11. if Collision(*q_temp_, q_i_*)
12. *q_temp_*←*q_i_*;
13. *Q*_2_.Add_Node(*q_temp_*);break
14. end if
15. end for
16. *Q*_2_.Add_Node(*q_n_*)
17. end while
18. for each node *q_k_* ∈ *Q*_2_
19. if Angle $(\vec{q_{k+1}q_{k}},\vec{q_{k+1}q_{k+2}})<\alpha_{\min}$
20. *Q*_2_ Insert_Node(*q_k_, q_insert_*, *q*_*k*+1_)
21. end if
22. end for
23. Return *Q*_2_

**Figure 1:**
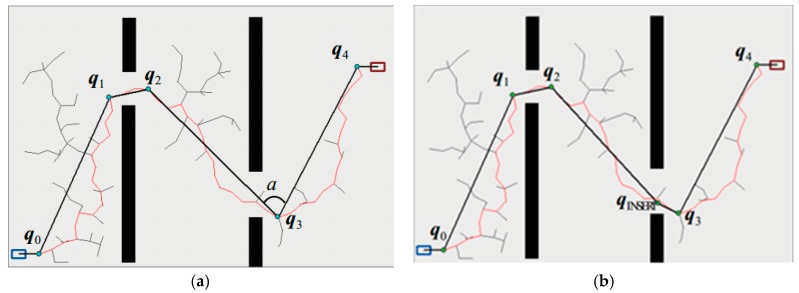
Pruning algorithm diagram. (**a**) Initial paths generated by the S-RRT; (**b**) Smooth paths using Function Pruning(*T*).

The improved RRT algorithm is defined as Smoothly RRT (S-RRT). The whole tree is pruned with Function Pruning(*T*) based on the maximum curvature constraint to delete unnecessary nodes and insert essential nodes. Then, the rest of the nodes are smoothed via using cubic *B* spline interpolation to generate an executable trajectory. The pruning function based on a maximum curvature constraint can be seen from line 5 to 23 of Function Pruning(*T*) in Trajectory Optimize(*T*). Firstly, a series of efficient path point sets $Q_{1}$ from the initial state to the target state are obtained from tree *T* of the above S-RRT. Then, the first path point of the initial state is connected with the subsequent path point. If the connecting lines have no intersection with obstacle space, path points can be deleted and their points will be connected by using one line, and so on. When a collision happens, the father node of the collision point is replaced as the new node and the above operations are executed again until the target state is reached. The path points obtained in the first step of post-processing are stored in $Q_{2}$. Next, according to the maximum curvature constraint, one path point is inserted based on $\alpha_{\min}$ in these lines, for which case the angle between the neighbor path segments in $Q_{2}$ is less than $\alpha_{\min}$. Thus, the sharp angles will flatten out so that the generated trajectory curvature is no more than the maximum curvature constraint with *B*-spline fitting. As is shown in Figure 1a, the red polyline represents the paths generated by the S-RRT algorithm. Since there is no intersection between the blue nodes $q_{0}$, $q_{1}$, $q_{2}$, $q_{3}$, and $q_{4}$ and the obstacle regions, they are able to be connected directly using lines so as to delete redundant nodes between them. $\alpha_{\min}$ denotes the minimum path angle with a default value of 90° in this paper. Considering $∠q_{2}q_{3}q_{4}<\alpha_{\min}$, the node $q_{insert}$ needs to be inserted based on $\alpha_{\min}$ to smooth the sharp angles to make $∠q_{insert}q_{3}q_{4}=\alpha_{\min}$, in which case $q_{2}$, $q_{3}$, and $q_{4}$ can also be connected directly. Finally, a set of flat path points are obtained, as shown in Figure 1b marked with green dots.

The *B* spline curve has the advantage with great continuity and locality [19,20] and has been widely applied in motion planning. Therefore, the *B* spline curve is utilized to fit path points which have been pruned beforehand to generate a smooth trajectory with a continuous curvature that can be followed by the manipulator later.

The expression with a *K*-order *B* spline curve is shown in Equation (1):

(1) $$C\left(u\right)=\sum_{i=0}^{n}N_{i,k}\left(u\right)\cdot P_{i}.$$

Here, $P_{i}$ is the control point, and the base function of the *B* spline curve can be obtained with the Cox–deBoor recursive relations:

(2) $$N_{i,0}\left(u\right)=\{\begin{array}{ll} 1 & u_{i}\leq u\leq u_{i+1}\\ 0 & otherwise \end{array}$$

(3) $$N_{i,k}\left(u\right)=\frac{u-u_{i}}{u_{i+k}-u_{i}}N_{i,k-1}\left(u\right)+\frac{u_{i+k+1}-u}{u_{i+k+1}-u_{i+1}}N_{i+1,k-1}\left(u\right).$$

For a *K*-order *B* spline curve and *n* control points, the node vector is $U=\left[u_{0},u_{1},u_{2},\cdots ,u_{m}\right]$ with m=n+K. The constraints of the initial and target state mean that the curves must go through the starting and target point as well as at a tangent to the control edges. To satisfy the constraints above, *K*-node vectors are used, namely, node vectors are satisfied with the following equations:

(4) $$\{\begin{array}{l} u_{0}=u_{1}=\cdots =u_{K}\\ u_{m-K}=u_{m-K+1}=\cdots =u_{m} \end{array}$$

## 3. Simulation and Experiment

### 3.1. Simulation in a Static Environment Based on MATLAB

**Figure 2:**
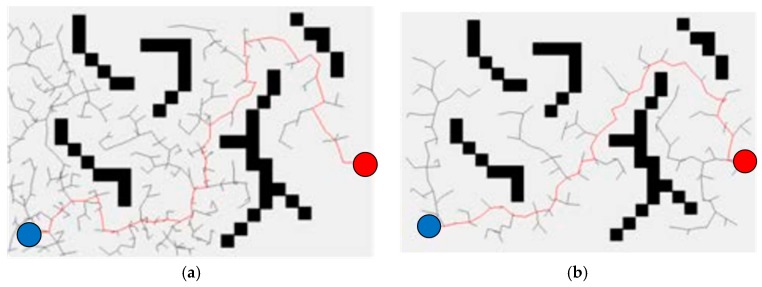

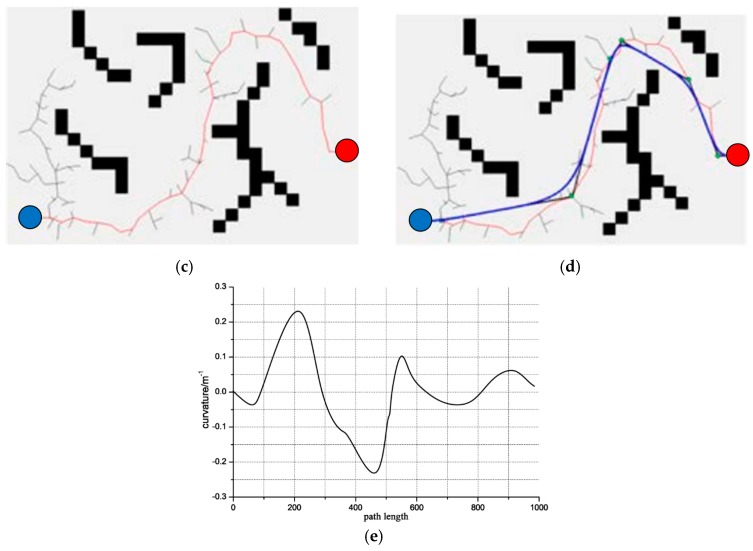
Comparison of the three algorithms and the result of the Smoothly Rapidly Exploring Random Tree (S-RRT) algorithm. (**a**) Random tree with Basic-RRT; (**b**) Random tree with Bi-RRT; (**c**) Random tree with S-RRT; (**d**) Random tree with optimized S-RRT; (**e**) Curvature variation map of the path generated by S-RRT.

Path planning for a robotic manipulator is high-dimensional manifold space planning. To prove the advantage and validity of the S-RRT algorithm, the simulation of two-dimensional (2D) path planning is carried out with static obstacles in MATLAB to compare it with the Basic-RRT and Bi-RRT algorithms. In this section, the manipulator is assumed to be the agent robot and the configuration space is the position in the scenario. The size of the whole state space is 600 × 400. The obstacle regions are black rectangular frames which are set up randomly. Path planning is performed via assigning the initial position (shown in the blue solid circle) and target position (shown in the blue solid circle) arbitrarily. The solution results of the same planning for Basic-RRT, Bi-RRT, and S-RRT are shown in the red broken lines of Figure 2a–c, respectively. Figure 2d gives the pruning result of the S-RRT algorithm (shown in green solid dots) and the final generated smooth path (shown in the blue Curve). The trees are pruned with the pruning algorithm to obtain sequence points without collision (shown in blue solid dots). Then, the sharp angles are processed by means of inserting nodes (shown in green solid dots). The curvature variation map of final path planning with S-RRT is shown in Figure 2e, from which it is shown that the curvature is continuous. Furthermore, considering the randomness of the RRT algorithm and an objective valuation of algorithm performance, 50 path planning experiments are carried out with three algorithms, respectively, in the same experiment scenario. Then, the average exploring time and sampling node number as well as successful exploring times are recorded, respectively, and are shown in Table 1.

From the related data in Figure 2 and Table 1 of the simulation experiment, the conclusion can be drawn that the exploring speed and exploring efficiency with S-RRT are improved better than those of Basic-RRT and Bi-RRT. Furthermore, the exploring paths are smoother and the generated path curvature is also continuous and executable, which meets the requirement of smooth and stable motion without shock for the manipulator in the process of obstacle avoidance.

### 3.2. Simulation Validation in a Dynamic Environment Based on an ROS

**Figure 3:**
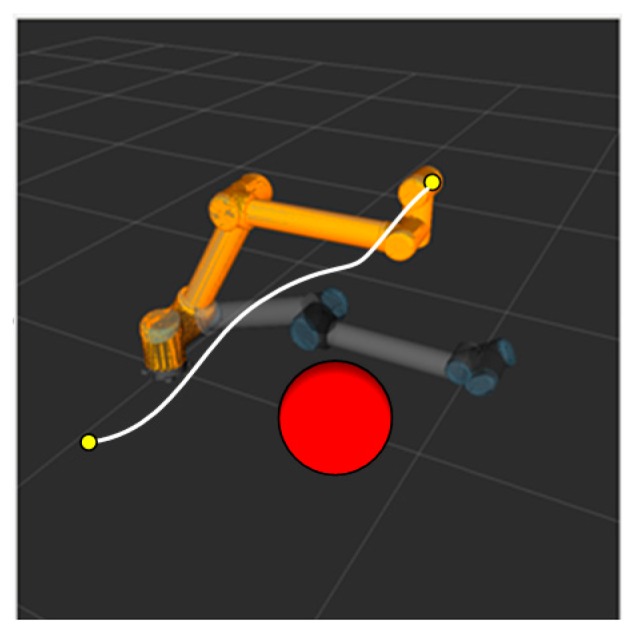
High-dimensional static RRT planning scenario.

The simulation experiment of dynamic obstacle avoidance with the RRT algorithm is performed in an ROS visualization tool called RViz. The marker in an ROS serves as a special Marker of the position of manipulator and the obstacle to show the motion planning of the RRT algorithm dynamically. The obstacles in a dynamic obstacle avoidance simulation exist in the form of markers in the ROS. The position of the obstacles can be controlled with a joystick to move up and down, front and back, and left and right as is shown in Figure 3. Additionally, the red ball represents a mobile obstacle with a radius of 0.1 m.

The yellow circle dots represent the initial position and the end position for the manipulator. The position and orientation of the initial pose and the target pose are the following (units: mm):

$$T_{start}=\left[\begin{array}{cccc} -0.38 & -0.13 & 0.92 & 622.57\\ 0.52 & 0.79 & 0.33 & -759.64\\ -0.76 & 0.60 & -0.23 & -330.72\\ 0 & 0 & 0 & 1 \end{array}\right]$$

$$T_{goal}=\left[\begin{array}{cccc} -0.30 & -0.79 & -0.54 & 445.41\\ -0.90 & -0.05 & 0.43 & -86.62\\ -0.32 & 0.61 & -0.73 & -866.29\\ 0 & 0 & 0 & 1 \end{array}\right]$$

**Figure 4:**
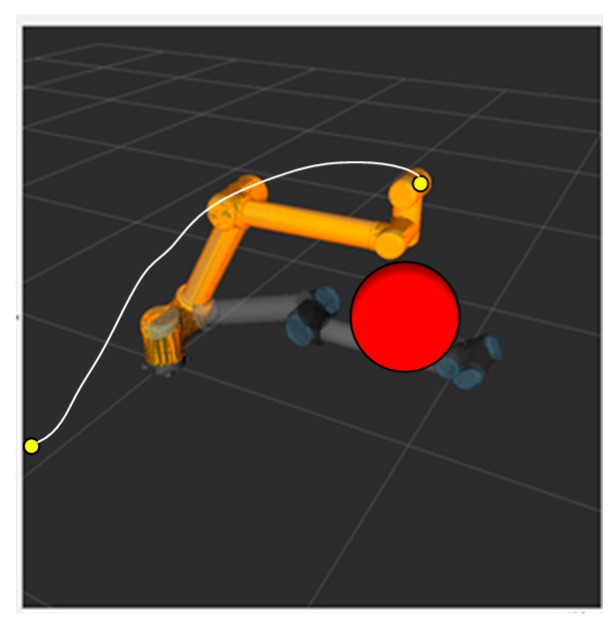
Replanned path after dynamic obstacle is moved.

When the obstacle is far away from the manipulator, motion planning cannot be affected. Figure 4 shows the path planned by the S-RRT algorithm when the obstacle is moved upward, from which we can see that the replanned path stays away from the obstacle without collision.

### 3.3. Experiment of Static Global Autonomous Obstacle Avoidance Path Planning

**Figure 5:**
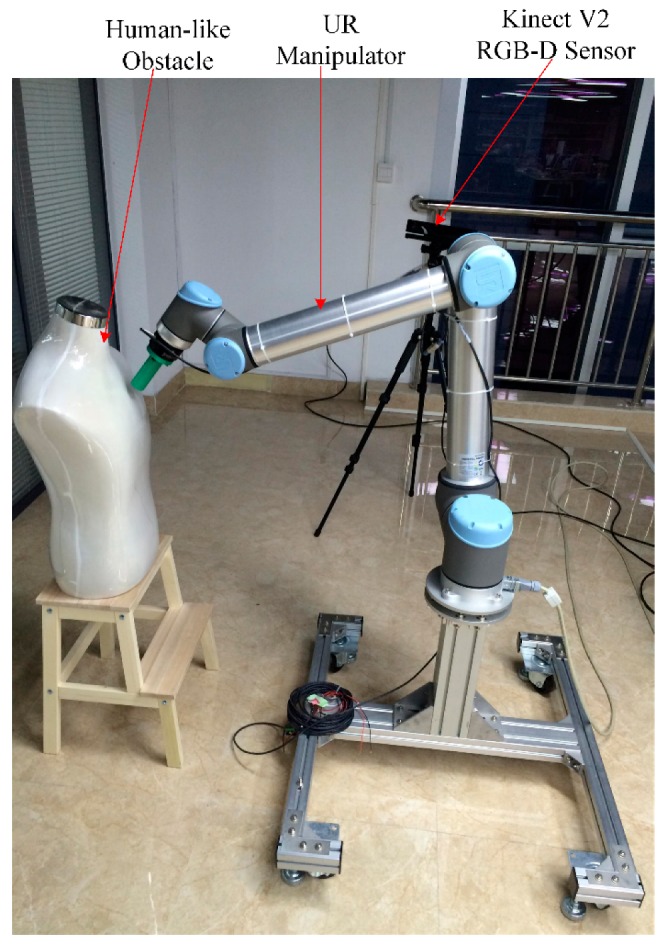
Experiment setup. UR represents Universal Robots.

To demonstrate the feasibility and effectiveness of the algorithm proposed by this paper, an experimental platform of path planning for a UR10 manipulator in a dynamic unstructured environment is constructed using the ROS Moveit as is shown in Figure 5. A Kinect V2 RGB-D sensor is placed on a tripod fixed in a special position to perceive the static and dynamic obstacles in the global environment. A human-like obstacle is also fixed in a certain position in the static environment. The depth map of the obstacle obtained with Kinect is firstly transformed into an octree map. The contour information of the obstacle can be obtained with the Point Cloud Library (PCL) process.

In this section, the feasibility and advantage of global obstacle avoidance with S-RRT in a static environment is validated in comparison with Basic-RRT and Bi-RRT. The initial pose and the target pose are the same as those of the simulation setup before.

**Figure 6:**
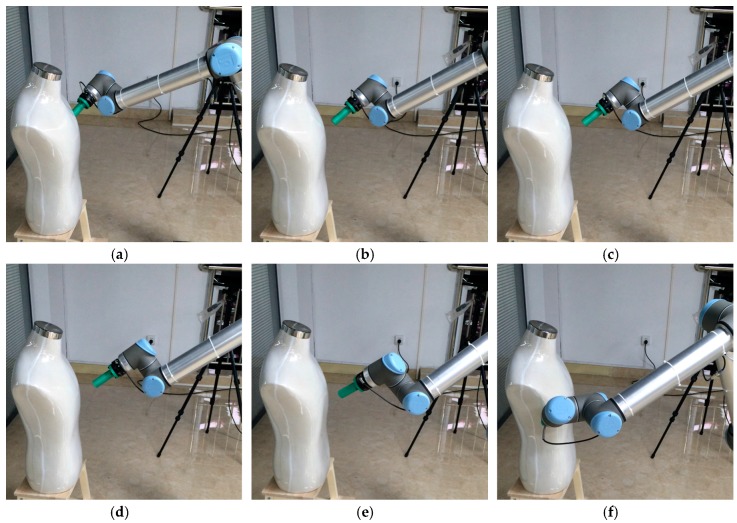
Path sequence with the S-RRT algorithm in static obstacle avoidance. (**a**–**f**) represent different states of the manipulator and static obstacle at each moment.

**Figure 7:**
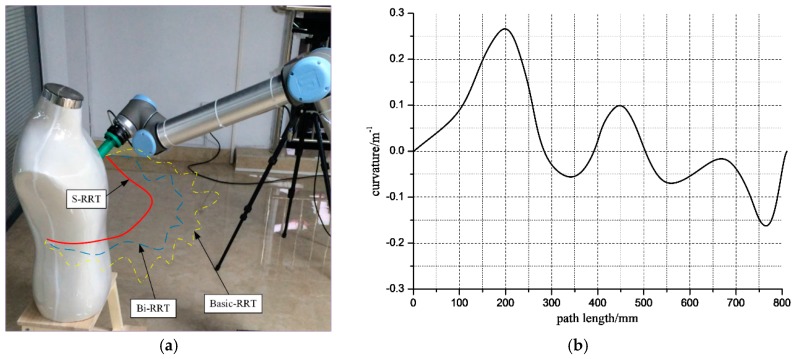
Comparison of the three algorithms and curvature variation with S-RRT. (**a**) Comparison of path planning with the three algorithms; (**b**) Variation of path curvature with path length.

**Figure 8:**
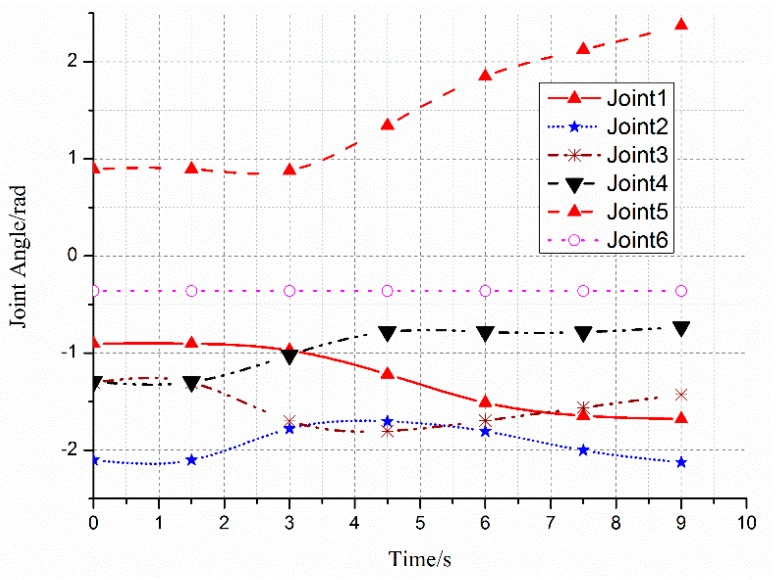
Variation of each joint angle during the process of obstacle avoidance.

The smooth path planned by the manipulator with the S-RRT algorithm can successfully avoid a static obstacle; the path sequence is shown in Figure 6. The comparison of the paths from the three algorithms is shown in Figure 7a and marked by a different color and line style. Additionally, Figure 7b also gives the S-RRT’s curvature variation with path length, from which we can see that the exploring path with S-RRT is the shortest and smoothest with a smooth curvature. The variations of each joint angle are shown in Figure 8.

In order to validate the algorithm proposed by this paper further, 20 experiments are performed and related data are recorded in Table 2, from which it is concluded that the exploring efficiency and successful rate of S-RRT are much higher than those of the other two algorithms.

### 3.4. Experiment of Dynamic Local Autonomous Obstacle Avoidance Path Planning

**Figure 9:**
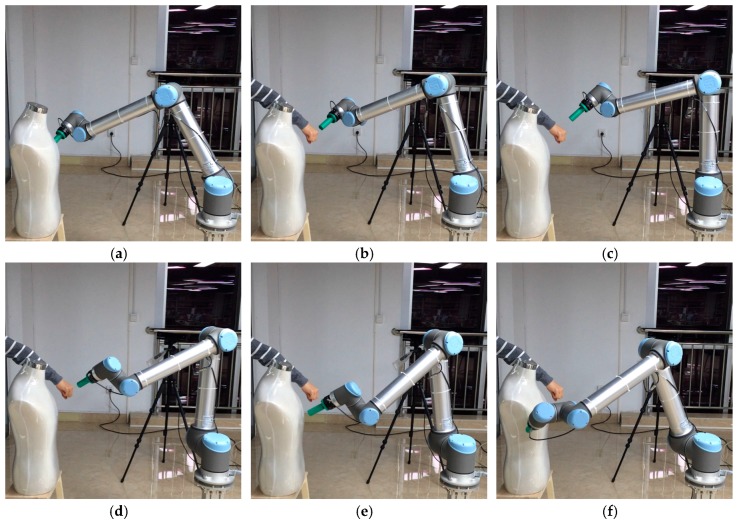
Path sequence with S-RRT algorithm during dynamic obstacle avoidance. (**a**–**f**) represent different states of the manipulator and dynamic obstacle at each moment

The capability for local planning obstacle avoidance with S-RRT is tested in this section. A human-like obstacle is fixed as a static obstacle. When the manipulator is tracking the path which has been planned before, suddenly, a dynamic obstacle is added to test the ability for dynamic obstacle avoidance, for example, a human arm is accessible in the manipulator’s workspace. The initial pose and the target pose are the same as those of the setup before. The path sequence with S-RRT algorithm during dynamic obstacle avoidance is shown in Figure 9.

**Figure 10:**
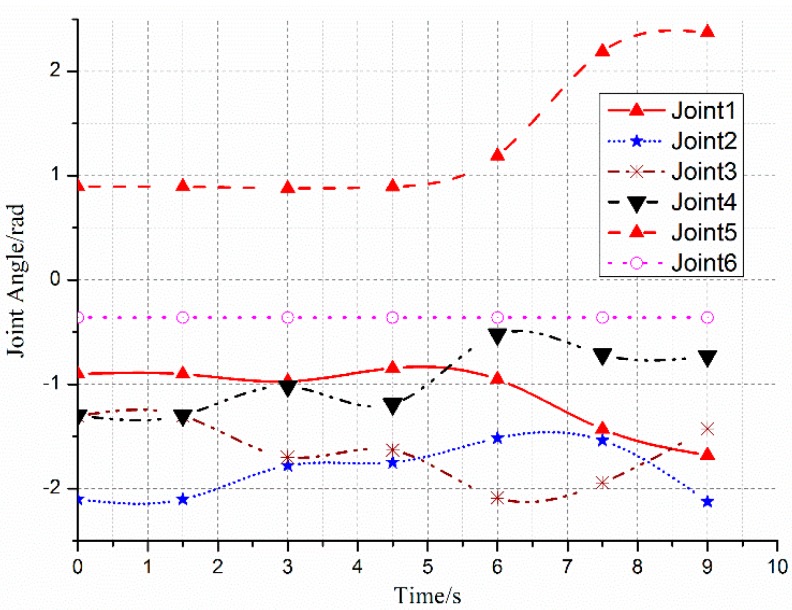
Variation of each joint angle during the process of dynamic obstacle avoidance.

**Figure 11:**
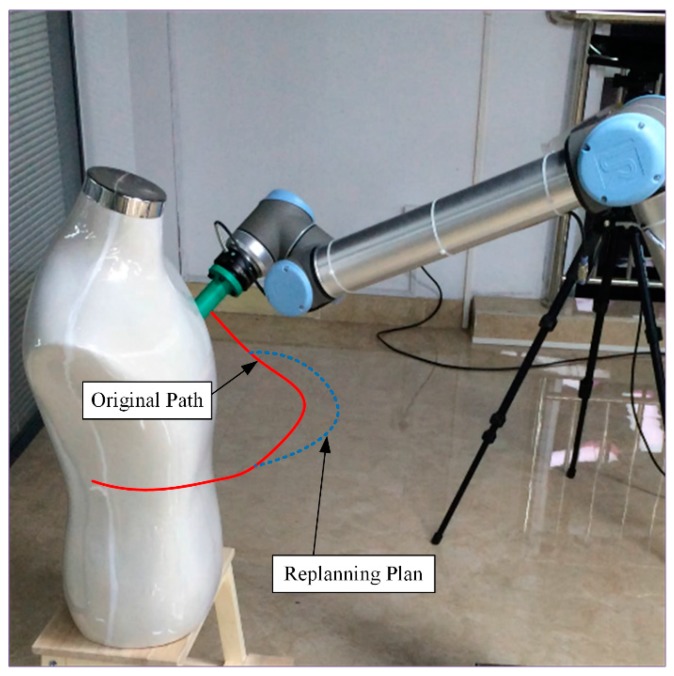
Replanned trajectory during the process of dynamic obstacle avoidance.

The variations of each joint angle of the replanned path during the process of dynamic obstacle avoidance are shown in Figure 10, from which we can see that the manipulator has changed the path at about 2.5 s and the process is smooth and without a shock. Figure 11 shows the replanned path when the obstacle suddenly appears. Furthermore, the first half-section and the last half-section are coincident after the obstacle suddenly appears. The manipulator can avoid the dynamic obstacle completely.

## 4. Conclusions and Future Work

The traditional RRT algorithm is improved upon with a target directional nodes extension and trajectory optimization based on the maximum curvature constraint in this paper. The exploring speed and efficiency with S-RRT are improved in comparison with Basic-RRT and Bi-RRT via a MATLAB static simulation, and the generated paths are much smoother with a continuous curvature in no more than one second. It is also demonstrated that the manipulator can not only avoid a static global obstacle, but also avoid a dynamic obstacle which may appear suddenly in a dynamic unstructured environment via an ROS simulation and a real experiment. The manipulator will replan the path quickly when encountering a new dynamic obstacle. After that, the manipulator will avoid the obstacle safely in a short time and continue to complete its assigned job. The whole obstacle avoidance process shows high autonomy and intelligence as well as the flexibility of the manipulator.

There are also limitations in this paper. A dynamic obstacle will be kept static after it appears suddenly and later tracking is not taken into consideration in this paper. Therefore, moving obstacle avoidance and tracking will be researched in a dynamic unstructured environment in the future.

## Figures and Tables

**Table 1:** Simulation comparison result of different algorithms.

50 Times Planning Experiments	Average Planning Time/ms	Average Sampling Nodes	Successful Times
Basic-RRT	403.5	752.6	42
Bi-RRT	186.75	351.8	50
S-RRT	79.4	172.3	50

**Table 2:** Simulation results with different algorithms.

20 Planning Experiments	Average Planning Time/ms	Average Sampling Nodes	Successful Times
Basic-RRT	986.5	1203.5	12
Bi-RRT	523.6	632.5	16
S-RRT	242.2	209.4	20
